# Inhaled corticosteroids, COPD, and the incidence of lung cancer: a systematic review and dose response meta-analysis

**DOI:** 10.1186/s12890-022-02072-1

**Published:** 2022-07-17

**Authors:** Tyler Pitre, Michel Kiflen, Terence Ho, Luis M. Seijo, Dena Zeraatkar, Juan P. de Torres

**Affiliations:** 1 Division of Internal Medicine, McMaster University, 1280 Main Street West, Hamilton, ON Canada; 2 Temerty School of Medicine, University of Toronto, Toronto, ON Canada; 3 Department of Respirology, St. Joseph’s Hospital, Hamilton, ON Canada; 4 Department of Pulmonary Medicine, Clínica Universidad de Navarra, Madrid, Spain; 5 Department of Bioinformatics, Harvard Medical School, Boston, MA USA; 6 Division of Respirology and Sleep Medicine, Queen’s University, Kingston, ON Canada; 7 Population Health Research Institute, McMaster University, Hamilton, Canada

**Keywords:** ICS, Lung cancer, COPD, Dose-response meta-analysis

## Abstract

**Background:**

There has been debate on whether inhaled corticosteroids (ICS) reduce the incidence of lung cancer amongst patients with Chronic Obstructive Lung Disease (COPD). We aimed to perform a systematic review and dose–response meta-analysis on available observational data.

**Methods:**

We performed both a dose response and high versus low random effects meta-analysis on observational studies measuring whether lung cancer incidence was lower in patients using ICS with COPD. We report relative risk (RR) with 95% confidence intervals (CI), as well as risk difference. We use the GRADE framework to report our results.

**Results:**

Our dose–response suggested a reduction in the incidence of lung cancer for every 500 ug/day of fluticasone equivalent ICS (RR 0.82 [95% 0.68–0.95]). Using a baseline risk of 7.2%, we calculated risk difference of 14 fewer cases per 1000 ([95% CI 24.7–3.8 fewer]). Similarly, our results suggested that for every 1000 ug/day of fluticasone equivalent ICS, there was a larger reduction in incidence of lung cancer (RR 0.68 [0.44–0.93]), with a risk difference of 24.7 fewer cases per 1000 ([95% CI 43.2–5.4 fewer]). The certainty of the evidence was low to very low, due to risk of bias and inconsistency.

**Conclusion:**

There may be a reduction in the incidence for lung cancer in COPD patients who use ICS. However, the quality of the evidence is low to very low, therefore, we are limited in making strong claims about the true effect of ICS on lung cancer incidence.

**Supplementary Information:**

The online version contains supplementary material available at 10.1186/s12890-022-02072-1.

## Introduction

Lung cancer remains one of the most common and deadliest malignancies in the world [1]. Despite significant research in therapies and screening, the prognosis for lung cancer remains poor [2]. Although reducing cigarette smoke is amongst the most effective interventions for reducing the risk of lung malignancy, for those patients with a significant previous or active smoking history, and those who develop Chronic Obstructive Lung Disease (COPD), the risk of lung malignancy remains high [3–5].

Significant interest and debate surround inhaled corticosteroids (ICS) and their potential role in the chemoprevention of lung cancer [6, 7]. A recent systematic review concluded that ICS use is associated with a decreased risk of lung cancer in obstructive lung disease [8]. Unfortunately, published cohorts are inconsistent and existing reviews have not addressed many important limitations of the evidence, such as risk of bias, nor have they assessed the certainty of evidence or explored a possible dose–response relationship.

Our objective is to perform a systematic review and meta-analysis, including a dose response analysis, on the effect of ICS for preventing lung malignancies in patients with COPD and to assess the certainty of evidence using the GRADE approach.

## Methods

We registered our protocol on Open Science Framework and present our results in accordance with the PRISMA guidelines: https://osf.io/jrdzp [9].

### Eligibility criteria

We included published and unpublished (abstracts, conferences, pre-prints) cohort studies that compared ICS with placebo/standard of care or different dosing regimens of ICS in patients with COPD. We also included mixed cohorts of asthma and COPD patients but excluded studies enrolling only asthma patients. We did not restrict study eligibility based on language or year of publication.

### Information sources

An experienced research librarian searched EMBASE, MEDLINE, Cochrane Controlled Register of Trials (CENTRAL), Web of Science, and MedRxiv databases from inception to January 2022. Additional file 1: Appendix A1 describes our search strategy.

### Data management and selection process

We uploaded citations to COVIDENCE, an online citation manager [10]. Pairs of reviewers, following calibration exercises to ensure sufficient agreement, worked independently and in duplicate to screen titles and abstracts of search records and subsequently the full texts of records determined potentially eligible at the title and abstract screening stage. Reviewers resolved discrepancies by discussion or, when necessary, by third party adjudication.

### Data collection process

Pairs of reviewers, following calibration exercises to ensure sufficient agreement, worked independently and in duplicate to collect data from eligible studies. Reviewers resolved discrepancies by discussion or, when necessary, by third party adjudication.

### Data items

We collected data on study characteristics (time and country of recruitment), patient demographics (age, sex), clinical characteristics (emphysema, bronchitis, mixed, COPD/asthma overlap), and factors potentially predictive of lung cancer (smoking status, duration of smoking, duration of COPD, history of cancer, long acting muscarinic antagonist/long acting beta agonist (LAMA/LABA) use, chronic antibiotics therapies, home oxygen therapy, non-invasive ventilation, and treatment with roflumilast, theophylline, oral steroids and type and dose of ICS). Our choice of co-variates was based on factors highly associated with the development of lung cancer [11].

### Outcomes and prioritization

We collected data on all-cause mortality, cancer-associated mortality, and serious adverse events. However, we only found data on the incidence of lung malignancy for analysis.

### Risk of bias

We assessed the risk of bias independently and in duplicate for each outcome using the risk of bias in non-randomised studies of interventions (ROBINS-I) tool [12]. We rated each outcome as either (1) low risk of bias, (2) moderate risk of bias, (3) serious risk of bias, and (4) critical risk of bias, across the following domains: bias due to confounding, bias in selection of participants into the study, bias in classification of interventions, bias due to deviations from intended interventions, bias due to missing data, bias in measurement of outcomes, and bias in selection of the reported result.

For studies to be rated as low risk of bias for confounding required at a minimum, adjustment for: age, sex, smoking (duration, pack years, quantity), COPD duration, socioeconomic status (employment, income, education), history of previous lung cancer, obesity, other lung disease (bronchiectasis, asthma, interstitial lung disease, obstructive sleep apnea), use of LAMA, LABA or both, treatment with oral corticosteroids and exposure to radon, radiation, or asbestosis. Additional file 1: Appendix A2 presents additional details on our assessment of risk of bias.

### Data synthesis

We report relative risk (RR) with 95% confidence intervals (CI) and risk differences per 1000 patients. To calculate risk differences, we used the baseline risk in a study we found most credible based on our assessment of risk of bias [13].

To compare the effects of lower versus higher doses of ICS and risk of lung cancer, we conducted a random-effects dose–response meta-analysis with the restricted maximum likelihood estimator (REML) using methods proposed by Greenland and Longnecker and Crippa and Orsini [14, 15]. Dose–response meta-analysis summarizes the quantitative relationship between doses of an exposure and the outcome across studies. We tested for nonlinearity using restricted cubic splines with knots at 10%, 50%, and 90% and a Wald-type test.

Because dose–response meta-analysis requires knowledge of the total number of participants or person-years, number of events, and mean or median dose across each dose category, not all studies were eligible for dose–response meta-analysis. Hence, we also present a random-effects meta-analysis with the REML estimator comparing the highest reported dose of ICS with the lowest reported dose across studies.

Where studies reported other types of ICS, we converted them to fluticasone equivalents. We used dose equivalents from data published by the Canadian Thoracic Society [16]. We made assumptions about dosing based on conversions and expert opinion from respirologist and consensus of the authors. For studies reporting doses per prescription, we assumed one prescription to be equivalent to 500 ug/day of fluticasone and two prescriptions to be equivalent to approximately 1000 ug/day. For studies reporting the dose of ICS as a range of values, we assigned the midpoint of upper and lower boundaries in each category as the average dose. If the highest or lowest category were open ended, we assumed that the open-ended interval is the same size as the most adjacent interval.

We evaluated heterogeneity in part by inspecting the I^2^ values: 0–39% as unimportant, 40–59% as moderate, 60–74% as substantial, and 75–100% as considerable heterogeneity. We performed a subgroup analysis for COPD only and asthma/COPD mixed cohorts. We also performed a meta-regression using reported sex as a moderator. No data was available on severity of COPD to perform subgroup analysis. We used the ICEMAN tool to assess the credibility of subgroups if the result was statistically significant [17].

We conducted all analysis using the meta, dosresmeta, and rcs packages in R, version 4.0.3 [14].

### Certainty of the evidence

We assessed the certainty of the evidence using the GRADE framework for observational studies and ROBINS-I [18, 19]. According to this approach, evidence starts at high certainty and may be further downgraded for risk of bias, inconsistency, indirectness, imprecision, or publication bias and may be upgraded for large effect, if suspected biases work against the observed direction of effect, or for dose–response gradient.

## Results

**Fig. 1:**
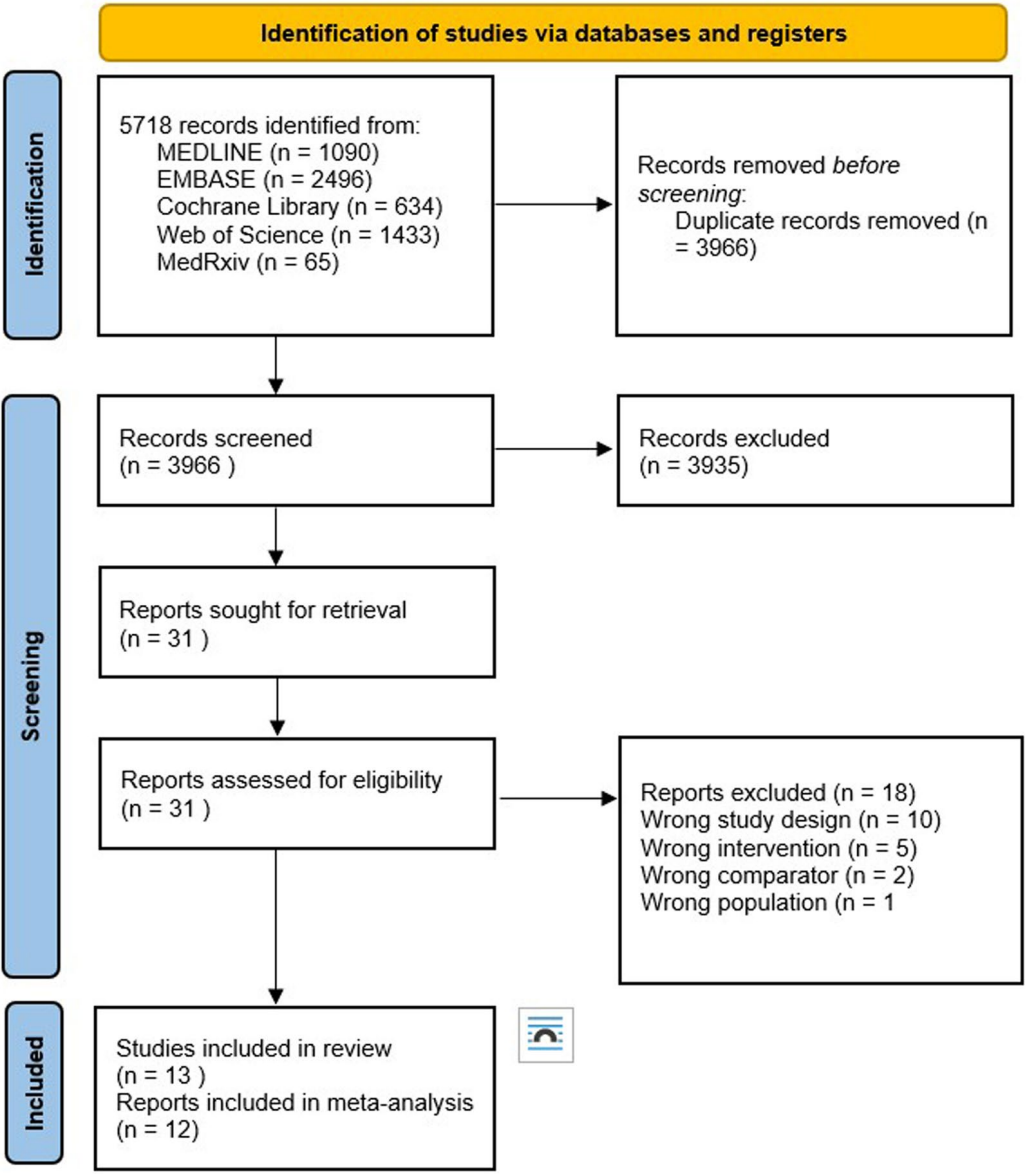
Flow diagram for inclusion and exclusion process

We identified 3964 citations and included thirteen studies with 268,363 patients. Figure 1 illustrates in more detail the inclusion and exclusion process. All but three studies reported only on COPD patients [20–22]. Studies reported on patients from seven different countries and three continents (Europe, Asia and North America) and collected data between 1966 and 2014. Studies reported primarily on elderly patients (median age: 66.4 years) and majority male. Two studies included only female patients [23, 24].

We identified three studies reporting on the patients from the Taiwan National Health Insurance Research Database, with overlapping patients [23–25], only one of which provided sufficient data for dose–response analysis. We included the study rated at lowest risk of bias in the highest versus lowest analysis [23].

Table 1 presents study characteristics [7, 21–24, 26–33].

**Table 1:** Study characteristics

Study	Country	Cohort database	Years included	Cohort size	Age	Male %	COPD %	Covariates adjusted	Range of doses
Husebo 2019	Norway	Bergen COPD Cohort Study between	2006–2009	712	61.3	57.4	100	Age, sex, smoking status, pack-years smoked, and body composition	0 to 1000 ug/day
Hyun 2012	South Korea	Korean National claims database	2007–2010	46,225	NR	NR	Unknown (COPD/Asthma)	NR	NR
Kiri 2009	United Kingdom	UK General Practice Research Database	1989–2003	7079	70.8	64.5	100	Age, sex duration of COPD, smoking, comorbidities including asthma, inhaler, other medications	NR
Lee 2018	South Korea	National Health Insurance Service–National Sample Cohort	2002–2013	1325	63.7	78	74 (COPD and Asthma)	Age, sex, pack years, BMI, income, comorbidities, duration of follow up	0–1000
Jian 2015	Taiwan	National Health Insurance Research Database (NHIRD)	2003–2010	3956	NR	87.4	NR (mixed; unspecified)	Sex, comorbidities, disease severity, previous lung cancer	0–2000 ug/day
Liu 2017	Taiwan	Taiwan's National Health Insurance (NHI) database	1997–2009	13,868	NR	0	100	Age, income, and comorbidities by cox regression mode	0–2000 ug/day
Parimon 2007	United States	Ambulatory Care Quality Improvement Project (ACQUIP)	1996–2001	10,474	64.1	97	100	Age, smoking status, smoking intensity, previous history of non–lung cancer malignancy, coexisting illnesses, and bronchodilator use	0 to > 1000 ug/day
Raymakers 2019	Canada	Medical Services Plan data	1997–2007	39,676	70.7	46.6	100	Age, sex, neighbourhood income quintile-based residence and British Columbia health authority (regional health service) in which the patient resided	0–640 ug/day
Sandelin 2018	Sweden	Department of Public Health and Caring Sciences	1999–2009	19,894	68.02	52.4	100	Age at COPD diagnosis, gender, asthma, education level, marital status, income prior to index, and time-dependent covariates medication and comorbidities	0–1000 ug/day
Sorli 2018	Norway	Nord-Trøndelag Health Study	1984–2008	4136	59.1	55.5	100	Sex, age, smoking pack years and FEV1% < 70	NR
Suissa 2020	Canada	Régie de l’Assurance Maladie du Québec	2000–2014	63,267	71.5	52.5	100	Age, sex, COPD hospitalisation and exacerbation in the year prior to cohort entry, as well as comorbidity at cohort entry, including cardiovascular and cerebrovascular diseases, diabetes, renal disease, other cancers (not lung), dementia and rheumatoid disease, among others, duration of ICS	0 to > 1000 ug/day
Wu 2016	Taiwan	Taiwan Health Insurance database	2003–2010	44,065	NR	69	100	Sex, age, medications, comorbidities, inpatient and outpatient visits for respiratory diseases, and urbanization	NR
Yang 2014	Taiwan	Taiwan Health Insurance database	1966–2011	13,686	NR	0	100	NR	NR

We contacted authors from three studies for number of participants and events across dose categories to facilitate dose–response meta-analysis [22, 26, 27, 31]. Two study authors provided us with this data [26, 31].

### Risk of bias

All studies were at serious risk of bias, mostly due to confounding and selection of the reported results. Most studies did not adjust for smoking (either duration or intensity), previous cancer diagnosis or relevant occupational (asbestos) or radon exposure. Nine studies were at risk of selection bias, as most did not account for duration of either COPD or ICS treatment. Two studies were at serious risk of bias due to classification of the intervention for not providing sufficient data. Eight studies were at moderate risk of bias due to deviations from the intended interventions since most studies were not able to confirm adherence to treatment. Two studies were at serious risk of bias due to missing data and two studies at moderate risk due to bias in the measurement of the outcome. All studies were at risk of bias in selection of the reported results for not having pre-specified protocols or statistical analysis plans. Table 2 summarizes our individual risk of bias judgements by cohort.

**Table 2:** Risk of bias assessments based on the ROBINS-I assessment tool

1st Author	Overall	Risk of bias (ROBINS-I)						
	Ranking	Bias due to confounding	Bias due to selection bias	Bias due to classification of intervention	Bias due to deviations from the intended intervention	Bias due to missing data	Bias in measurement of outcome	Bias in selection of the reported results
Yang	Serious	Serious	Serious	Low	Moderate	Low	Low	Serious
Parimon	Serious	Serious	Serious	Low	Low	Low	Low	Serious
Kiri	Serious	Serious	Low	Low	Low	Low	Low	Serious
Liu	Serious	Serious	Serious	Low	Moderate	Low	Low	Serious
Sandelin	Serious	Serious	Serious	Low	Moderate	Low	Low	Serious
Sorli	Serious	Serious	Serious	Low	Moderate	Low	Low	Serious
Raymakers	Serious	Serious	Low	Low	Moderate	Low	Low	Serious
Husebo	Serious	Serious	Serious	Low	Moderate	Low	Low	Serious
Suissa	Serious	Serious	Low	Low	Low	Low	Low	Serious
Lee	Serious	Serious	Low	Low	Moderate	Low	Low	Serious
Yang	Serious	Serious	Serious	Serious	Serious	Serious	Moderate	Serious
Wu	Serious	Serious	Serious	Low	Moderate	Low	Low	Serious
Hyun	Serious	Serious	Serious	Serious	Serious	Serious	Moderate	Serious
Jian	Serious	Serious	Low	Moderate	Moderate	Low	Low	Serious

### Dose response meta-analysis: incidence of lung cancer

Seven studies could be included in the dose–response meta-analysis. Our dose–response suggested a reduction in the incidence of lung cancer for every 500 ug/day of fluticasone equivalent ICS (RR 0.82 [95% 0.68–0.95]). Using a baseline risk of 7.2%, we calculated risk difference of 14 fewer cases per 1000 ([95% CI 24.7–3.8 fewer]). Similarly, our results suggested that for every 1000 ug/day of fluticasone equivalent ICS, there was a larger reduction in incidence of lung cancer (RR 0.68 [0.44–0.93]), with a risk difference of 24.7 fewer cases ([95% CI 43.2–5.4 fewer]).

**Fig. 2:**
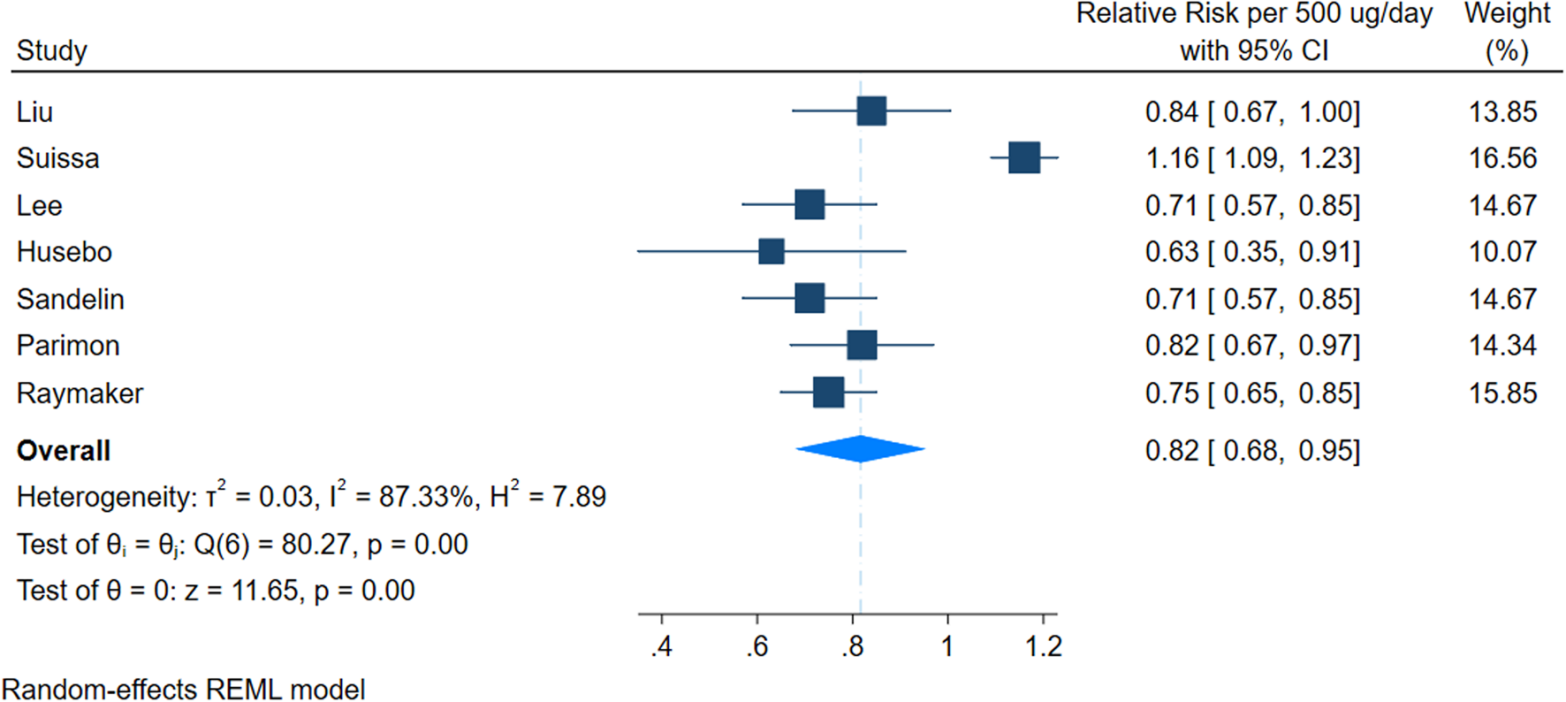
Dose response meta-analysis per 500 µg/day

**Fig. 3:**
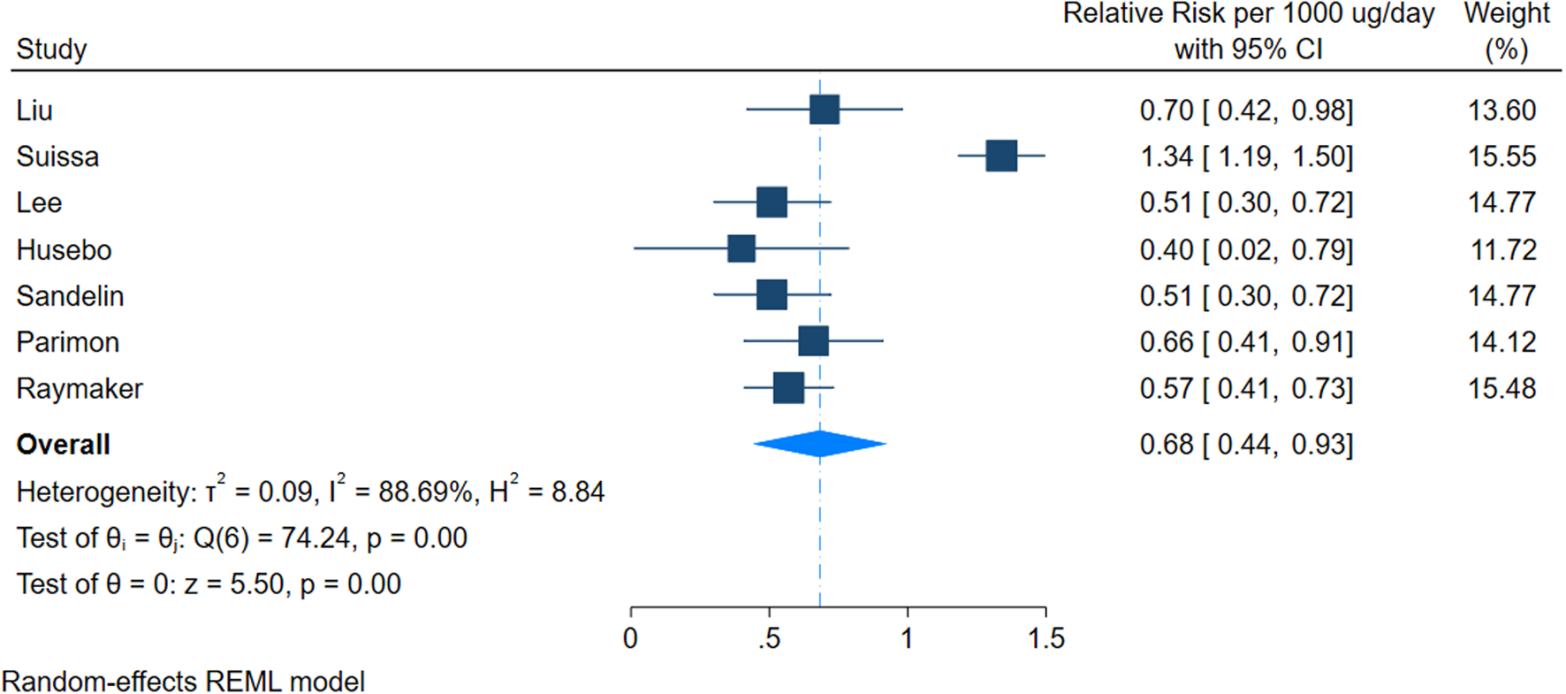
Dose response meta-analysis per 1000 µg/day

The certainty of evidence was very low due to risk of bias and inconsistency. Figure 2 and Fig. 3 present the results. We did not find evidence of non-linearity in the analysis (*p* = 0.16).

### High versus low: incidence of lung cancer

Eleven studies could be included in the meta-analysis comparing highest versus lowest ICS exposure and lung cancer. Our meta-analysis suggested higher dose ICS to reduce the risk of lung cancer (RR 0.70 [95% 0.52–0.96]), but there was substantial heterogeneity (I^2^ = 87.57%). Using a baseline risk of 7.2%, we calculated a risk difference of 19.8 fewer cases per 1000 ([95% CI 35–2.9]).

**Fig. 4:**
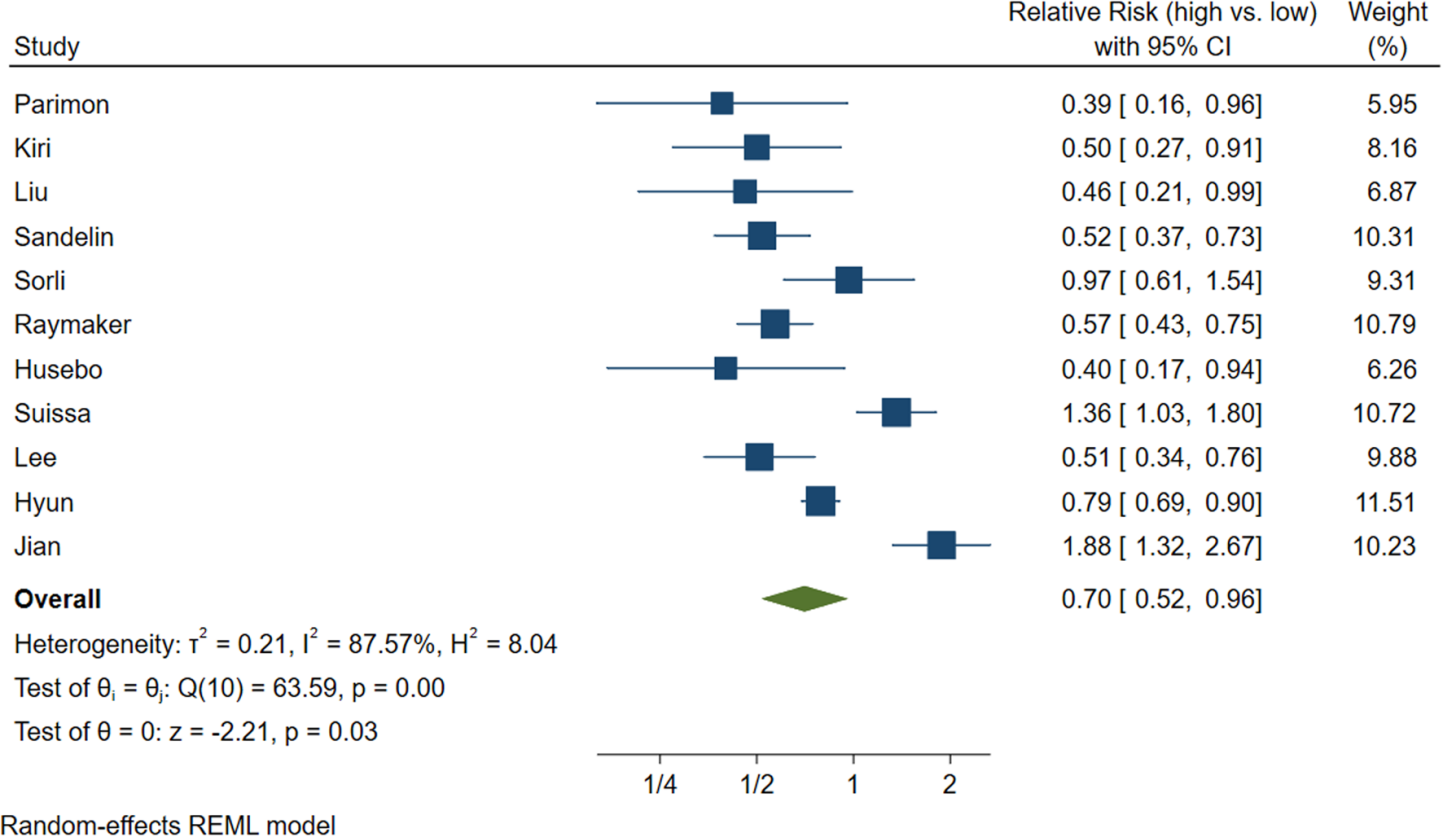
High versus low inhaled corticosteroids meta-analysis

**Fig. 5:**
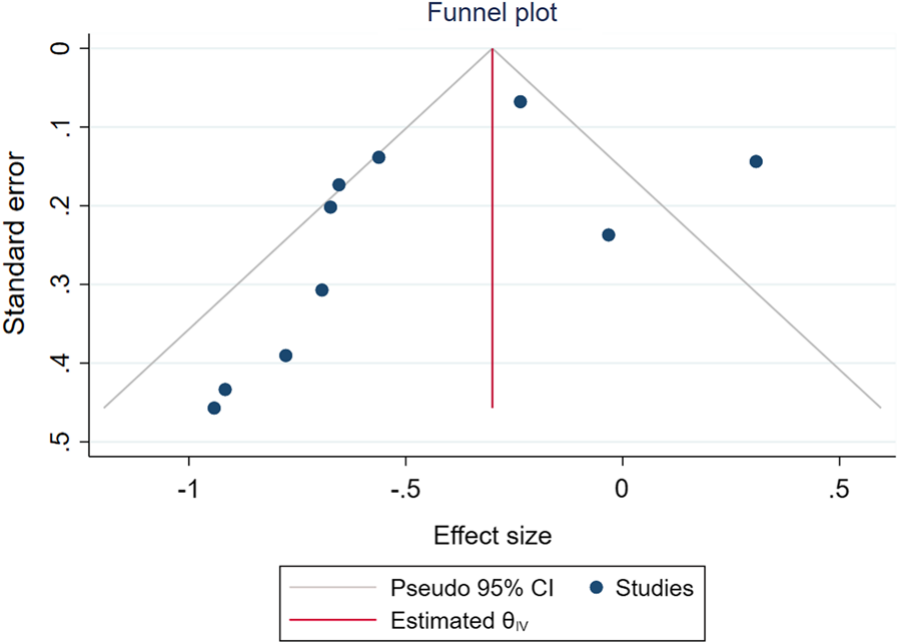
Funnel plot for high versus low inhaled corticosteroids

We rated this as very low certainty due to risk of bias and inconsistency. Figure 4 presents more details on the high versus low ICS studies. We did not detect evidence of publication bias using inspection of the funnel plot and Egger’s statistical test (*p* = 0.07) (Fig. 5).

### Subgroup analysis

We did not find a statistically significant difference in results between mixed cohorts of COPD and asthma versus COPD only cohorts (*p* = 0.36), nor was sex a statistically significant moderator in a meta-regression model (*p* = 0.5).

### All-cause mortality, cancer-associated mortality, and serious adverse events

Data was unavailable for these outcomes.

## Discussion

### Main findings

Our review presents a comprehensive and rigorous analysis of the evidence addressing the relationship between ICS treatment and lung cancer in COPD patients. We not only explore evidence of a dose–response relationship, but we summarize and appraise the quality of the evidence using the GRADE approach.

The present meta-analysis found that there may be a dose-dependent association between ICS treatment in COPD patients and a reduction in the incidence of lung cancer but the evidence is very uncertain. The risk of bias of the studies, for example, was high, primarily due to potential for confounding bias. Most cohorts were unable to adjust for important predictors of lung cancer, including smoking, or adherence to ICS treatment. There was considerable heterogeneity across studies highlighting important differences between the included cohorts. Therefore, we are limited in our conclusions with regards to the true effect of ICS on lung cancer incidence.

### In relation to other findings

The use of ICS as lung cancer chemoprevention has been debated. There have been no randomized trials designed to investigate the impact of ICS on lung cancer incidence. However, three trials randomized patients to ICS in other contexts and reported on the incidence of lung cancer, showing no benefit, though they were all underpowered to answer this question [34–37].

Two previous systematic reviews and meta-analyses compared high versus low ICS in COPD patients that reported results that differed from our analysis [38, 39]. Both reviews compared high versus low ICS without a dose response analysis. However, there are substantial limitations that circumscribe their analysis and significantly hinder their conclusions about the effectiveness ICS in reducing the incidence of lung cancer in COPD patients. First, neither reviews use a system for rating the certainty of the evidence such as GRADE, making the results less meaningful to evidence users. Second, the reviews did not assess the risk of bias of the studies using a recommended risk of bias tool for observational data. For example, both reviews provide only quality ratings for studies, not specific risk of bias assessments. Third, the reviews did not present absolute effects. Fourth, the reviews did not include as many cohorts as the present meta-analysis. Both previous meta-analyses conclude that ICS is effective at reducing lung cancer incidence. Our analysis shows that there is very low certainty evidence for this conclusion and given the significant inconsistency and risk of bias, we caution making such strong claims.

The inconsistency of the data is of particular concern. Two studies showed harm with escalating doses of ICS in COPD patients and one trial showed no effect [7, 22]. One of these studies included a large number of asthma patients, which is typically thought to overestimate the effect of ICS on lung cancer mortality, but instead showed an increased risk of lung cancer incidence.

### Limitations

The strengths of our review include use of two meta-analytic methods, as well as rigorous and state-of-the-art methods for rating the risk of bias assessment and the certainty of the evidence [18].

Important limitations of our dose response analysis include our estimation of ICS doses. We made crude assumptions about fluticasone equivalence when not directly reported and cannot be certain of the level of adherence to ICS treatment in most studies. Furthermore, we were unable to include all studies in the dose response analysis, potentially obfuscating the true dose response effect. For example, one study that showed a negative relationship between ICS and lung cancer could not be included in the dose–response meta-analysis [22].

Another limitation is that we included three mixed asthma/COPD cohorts. However, this was expected to overestimate the effect of ICS on reducing lung cancer incidence, but we found no difference in subgroups. Furthermore, current evidence suggests that COPD is often underdiagnosed and over treated. Ongoing modifications to established guidelines recommending ICS treatment for different COPD stages and phenotypes also make the study of ICS effects in COPD a constantly moving target. The clinical need for well designed, adequately powered, randomized trials of lung cancer chemoprevention using ICS, remains unmet. Finally, there were limited data to perform subgroup analysis, including underlying disease severity (GOLD classifications), COPD phenotypes and lung function. Existing evidence linking COPD severity to varying degrees of risk for lung cancer suggests that not all COPD patients may have comparable risks of malignancy.

## Conclusion

ICS treatment may reduce the incidence of lung cancer in COPD patients, but the certainty of evidence is very low. However, available data originates from cohorts at serious risk of bias, plagued by inconsistency and heterogeneity. High quality cohort studies or randomized controlled trials are needed to improve the certainty of the evidence.

## Supplementary Information

**Additional file 1**. **A1.** Search strategy for Medline 2. **A2.** Risk of bias tool (ROBINS-I).

## Data Availability

The datasets generated and analysed during the current study will be available in the Open Science Framework repository at https://osf.io/jrdzp/ upon publication.
