# Supplementary material for: Inhaled corticosteroids, COPD, and the incidence of lung cancer: a systematic review and dose response meta-analysis
Source: BMC Pulm Med. 2022 Jul 17;22:275. doi: 10.1186/s12890-022-02072-1 (PMC9290283; doi:10.1186/s12890-022-02072-1)
Supplement: Supplementary file 1 — Additional file 1. A1. Search strategy for Medline 2. A2. Risk of bias tool (ROBINS-I). [file 12890_2022_2072_MOESM1_ESM.pdf]

Table of contents

A1. Search strategy for Medline 2

A2. Risk of bias tool (ROBINS-I) 4

A1. Search strategy for Medline

Database: OVID Medline Epub Ahead of Print, In-Process & Other Non-Indexed Citations, Ovid MEDLINE(R) Daily and Ovid MEDLINE(R) 1946 to Jan 18, 2022
Search Strategy:
----
1 exp Pulmonary Disease, Chronic Obstructive/ (61879)
2 COPD.mp. (52866)
3 (obstructive adj3 pulmonary).mp. [mp=title, abstract, original title, name of substance word, subject heading word, floating sub-heading word, keyword heading word, organism supplementary concept word, protocol supplementary concept word, rare disease supplementary concept word, unique identifier, synonyms] (73372)
4 or/1-3 (95527)
Annotation: COPD concept
5 Administration, Inhalation/ (32762)
6 adrenal cortex hormones/ or exp glucocorticoids/ (260640)
7 exp Steroids/ (889614)
8 6 or 7 (971439)
9 5 and 8 (8896)
10 ICS.mp. (10052)
11 ((inhale* or inhalat* or vapor or vapour) adj3 (steroid* or corticosteroid)).mp. [mp=title, abstract, original title, name of substance word, subject heading word, floating sub-heading word, keyword heading word, organism supplementary concept word, protocol supplementary concept word, rare disease supplementary concept word, unique identifier, synonyms] (7022)
12 (budesonide or fluticasone or beclomethasone or ciclesonide or flunisolide or mometasone or triamcinolone).mp. [mp=title, abstract, original title, name of substance word, subject heading word, floating sub-heading word, keyword heading word, organism supplementary concept word, protocol supplementary concept word, rare disease supplementary concept word, unique identifier, synonyms] (27008)
13 or/9-12 (41631)
Annotation: inhaled corticosteroids
14 4 and 13 (3627)

Annotation: COPD and inhaled corticosteroids
15 exp Lung Neoplasms/ (253966)
16 ((lung or pulmonary) adj3 (cancer or neoplasm*)).mp. [mp=title, abstract, original title, name of substance word, subject heading word, floating sub-heading word, keyword heading word, organism supplementary concept word, protocol supplementary concept word, rare disease supplementary concept word, unique identifier, synonyms] (303006)
17 15 or 16 (315763)
18 14 and 17 (57)
19 ("17185647" or "18793832" or "28412726" or "29922050" or "29427221" or "30956205" or "31128615" or "31744837" or "29943812").ui. (9)
20 18 and 19 (9)
21 exp Mortality/ or mortality.mp. (1407100)
22 ae.fs. (1868453)
23 adverse event*.mp. (191446)
24 17 or 21 or 22 or 23 (3407849)
25 14 and 24 (1446)
26 prognosis/ (561423)
27 exp risk/ (1318877)
28 exp PROBABILITY/ (1517826)
29 exp Regression Analysis/ (451214)
30 "analysis of variance"/ or multivariate analysis/ (343660)
31 exp Epidemiologic Studies/ (2860822)
32 (prognosis or prognostic or predict* or risk*).mp. (4963271)
33 ((univariate or covariate or variance or covariance or multivariate or regression or adjusted or unadjusted or logistic or diagnostic) adj2 (analys* or model*)).mp. (1263540)
34 (logistic adj2 regress*).mp. (345699)
35 ((cohort or observational) adj3 (study or studies or analy*)).mp. [mp=title, abstract, original title, name of substance word, subject heading word, floating sub-heading word, keyword heading word, organism supplementary concept word, protocol supplementary concept word, rare disease supplementary concept word, unique identifier, synonyms] (701604)

36 (longitudinal or retrospective or cross sectional or prospective).mp. [mp=title, abstract, original title, name of substance word, subject heading word, floating sub-heading word, keyword heading word, organism supplementary concept word, protocol supplementary concept word, rare disease supplementary concept word, unique identifier, synonyms] (2717171)
37 (Follow up adj (study or studies)).tw. (52750)
38 questionnaire$.mp. or Questionnaires/ (851506)
39 ep.fs. (1910507)
40 or/26-39 (8478904)
41 25 and 40 (1054)
42 18 or 41 (1064)

A2. Risk of bias tool (ROBINS-I)

Domains	Low	Moderate	Serious	Critical
Confounding	Study adjusts for: age, sex, smoking (duration, pack years, quantity), socioeconomic status (either one of: employment, income, education), obesity, other lung disease (bronchiectasis, asthma, interstitial lung disease, obstructive sleep apnea), use of LAMA, LABA, or both, oral corticosteroids and exposure to radon,	Study adjusts for age, sex, smoking (duration, pack years, quantity), socioeconomic status (either one of: employment, income, education), and exposure to radon, radiation, and asbestos.	Study adjusts for age, sex, smoking (duration, pack years, quantity)	Study does not adjust for either age, sex, or smoking

	radiation, and asbestos.<br><br>All confounders have been appropriately measured and accounted for either in the design or in the analysis.			
**Selection bias**	The analysis excludes patients who used inhaled corticosteroid therapy before diagnosis of COPD and patients who had a history of lung cancer before diagnosis of COPD. The analysis controls for the duration between COPD diagnosis and intitiaton of medication or eligibility for medication.		The analysis excludes patients who used inhaled corticosteroid therapy before diagnosis of COPD and patients who had a history of lung cancer before diagnosis of COPD. The analysis does not control for the duration between COPD diagnosis and intiation of medication or eligibility for medication.	The analysis includes patients who used inhaled corticosteroid therapy before diagnosis of COPD and/or patients who had a history of lung cancer before diagnosis of COPD.
**Classification of intervention**	Classification of intervention is based on prescriptions.			
**Deviation from intended intervention**	There is evidence of very high adherence to the assigned intervention and dose. The study investigators confirmed	There is potential for non-adherence of sufficiently great magnitude to impact results. The study investigators did not confirm	There is evidence of non-adherence or possibility of non-adherence such that the study results may have been impacted.	There is evidence of non-adherence of sufficiently great magnitude to majorly impact results.

	adherence.	adherence.		
**Missing data**	<3% missing data Proportions of and reasons for missing participants are similar across interventions and doses	3-<5% missing data Proportion of missing data and reasons for missingness differ slightly across interventions and doses	5-<15% missing data Proportion of missing data and reasons for missingness differ substantially across interventions and doses	15% or more missing data Proportion of missing data or reasons for missingness differ critically across interventions and doses
**Measurement of outcome**	All-cause mortality, lung cancer, lung cancer mortality Adverse events if healthcare providers were blinded to corticosteroid use and dose.		Adverse events if healthcare providers were not blinded to corticosteroid use and dose.	
**Selective reporting**	Results that are conducted and analyzed according to a prespecified protocol and statistical analysis plan with no deviations from the protocol and statistical analysis plan.		Studies that are not conducted or analyzed according to a prespecified protocol or statistical analysis plan.	
